# High-Pressure Synthesis of Pnictogen Nitrides

**DOI:** 10.1021/acs.accounts.5c00309

**Published:** 2025-09-26

**Authors:** Matteo Ceppatelli, Manuel Serrano-Ruiz, Marta Morana, Kamil Dziubek, Demetrio Scelta, Roberto Bini, Maurizio Peruzzini

**Affiliations:** † ICCOM-CNR, Institute of Chemistry of OrganoMetallic Compounds, National Research Council of Italy, Via Madonna del Piano 10, I-50019 Sesto Fiorentino, Firenze, Italy; ‡ LENS, European Laboratory for Non-linear Spectroscopy, Via N. Carrara 1, I-50019 Sesto Fiorentino, Firenze, Italy; § Dipartimento di Scienze della Terra, 9300 Università degli Studi di Firenze, Via G. La Pira 4, I-50121 Firenze, Italy; ∥ Institut für Mineralogie und Kristallographie, Universität Wien, Josef-Holaubek-Platz 2, A-1090 Wien, Austria; ⊥ Dipartimento di Chimica “Ugo Schiff”, 226476 Università degli Studi di Firenze, Via della Lastruccia 3, I-50019 Sesto Fiorentino, Firenze, Italy

## Abstract

Nitrides represent a class of chemical compounds of high scientific and technological relevance. Nevertheless, due to the challenging synthetic conditions, essentially related to the stability of the N_2_ molecule, nitrides have remained largely unexplored compared with the corresponding oxides.

The laser-heated diamond anvil cell (LH-DAC), providing access to the GPa pressure range and temperatures as high as several thousands of K, has dramatically changed the rules of the game, unveiling thermodynamic conditions in which N_2_ becomes unstable and polymerizes into extended crystalline phases. A variety of N-compounds have been indeed synthesized by laser-heating the corresponding elements in a N_2_ environment under high-pressure (HP) conditions.

Nevertheless, only recently group 15 elements heavier than N have been targeted by this method, and excluding α-P_3_N_5_ and γ-P_3_N_5_, crystalline pnictogen nitrides have remained essentially unknown.

Since the discovery of phosphorene, while the quest for 2D materials has raised the interest for group 15 xenes and their N-doping as a key development, the inherent tendency of pnictogens to adopt crystalline layered structures persisting at high pressure has increased focus on binary N-compounds with heavier pnictogens, with implications for fundamental chemistry and potential applications.

In this scenario, the discovery of the pseudo simple-cubic (p-sc) structure in the phase diagram of P, and, a few years later, the high-pressure–high-temperature (HP-HT) synthesis of PH_3_ from the elements and the discovery of the crystalline van der Waals (vdW) compound (PH_3_)_2_H_2_ have further reconnected the HP behavior of P to that of lighter N.

These experimental studies, highlighting consistency in the structural and reactive properties of group 15 elements at high pressure, together with additional theoretical and computational insights, have opened new perspectives and motivated further investigations about the existence of crystalline pnictogen nitrides.

Indeed, not only α- and γ-P_3_N_5_, but also three other crystalline polymorphs of phosphorus nitride (δ-P_3_N_5_, PN_2_, and α′-P_3_N_5_), have been synthesized by the direct HP-HT chemical reaction of P and N_2_ in a LH-DAC.

Moving down in group 15, the same method has led to the discovery of the first crystalline nitrides of arsenic (AsN) and antimony (Sb_3_N_5_), whose existence has always represented an open question in inorganic chemistry, and to the structural characterization of two crystalline polymorphs of bismuth nitride (BiN).

This Account provides an overview of the recent progress in the high-pressure and high-temperature synthesis of crystalline pnictogen nitrides, demonstrating the effective activation of a direct chemistry between N and heavier pnictogens. The presented results mark fundamental advancements in the chemistry of group 15 elements and pioneer the discovery of new advanced pnictogen-based materials of energetic and technological relevance, potentially recoverable under ambient conditions as stable or metastable systems.

## Graphical abstract

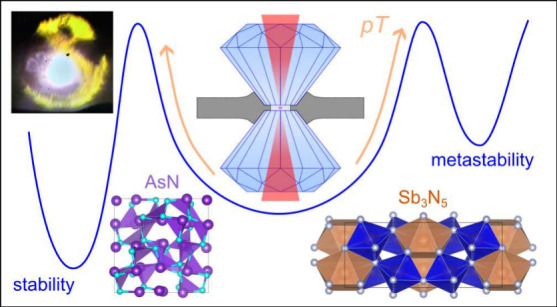

## 1 Key References

-

Scelta, D. ; Baldassarre, A. ; Serrano-Ruiz, M. ; Dziubek, K. ; Cairns, A. B. ; Peruzzini, M. ; Bini, R. ; Ceppatelli, M. Interlayer Bond Formation in Black Phosphorus at High Pressure. Angew. Chem. Int. Ed. 2017, 56, 14135–14140 10.1002/anie.201708368PMC583688528940812.^1^ *Discovery of the pseudo simple-cubic (p-sc) structure in the phase diagram of phosphorus. Solving anomalies and reconciling the high-pressure structural behavior of P with respect to heavier pnictogens.*
-

Ceppatelli, M. ; Scelta, D. ; Serrano-Ruiz, M. ; Dziubek, K. ; Izquierdo-Ruiz, F. ; Recio, J. M. ; Garbarino, G. ; Svitlyk, V. ; Mezouar, M. ; Peruzzini, M. ; Bini, R. High-Pressure and High-Temperature Chemistry of Phosphorus and Nitrogen: Synthesis and Characterization of α- and γ-P_3_N_5_
. Inorg. Chem. 2022, 61, 12165–12180 35881069 10.1021/acs.inorgchem.2c01190PMC9374155.^2^ *Synthesis of α- and γ-P*_3_ *N*_5_ *from P and N*_2_. *Identification of α-P*_3_ *N*_5_ *as intermediate step toward γ-P_3_ *N*_5_, with 4 → 5 increase of the coordination number of P by N*. *Characterization of γ-P*_3_ *N*_5_ *during room T compression and observation of metastable α-P*_3_ *N*_5_.
-

Ceppatelli, M. ; Scelta, D. ; Serrano-Ruiz, M. ; Dziubek, K. ; Morana, M. ; Svitlyk, V. ; Garbarino, G. ; Poręba, T. ; Mezouar, M. ; Peruzzini, M. ; Bini, R. Single-Bonded Cubic AsN from High-Pressure and High-Temperature Chemical Reactivity of Arsenic and Nitrogen. Angew. Chem. Int. Ed. 2022, 61, e202114191 10.1002/anie.202114191PMC930422734797602.^3^ *Discovery of crystalline AsN: synthesis and XRD characterization of the first crystalline nitride of arsenic. Key-role of the electron lone pairs.*
-

Ceppatelli, M. ; Serrano-Ruiz, M. ; Morana, M. ; Dziubek, K. ; Scelta, D. ; Garbarino, G. ; Poręba, T. ; Mezouar, M. ; Bini, R. ; Peruzzini, M. High-pressure and high-temperature synthesis of crystalline Sb_3_N_5_
. Angew. Chem. Int. Ed. 2024, 63, e202319278.10.1002/anie.20231927838156778 ^4^ *Discovery of crystalline Sb*_3_ *N_5_: the long sought-after synthesis of a crystalline nitride of antimony and its structural characterization by single-crystal synchrotron XRD.*

## 2 Introduction

Crystalline nitrides and high-nitrogen content materials have always fascinated chemists, physicists, and materials scientists for their mechanical, electronic, and energetic properties. Nevertheless, the difficulty of their synthesis has always represented a challenging limitation to their study.^5,6^ In this regard, a great benefit is offered by the application of pressure. Indeed, a plethora of new binary compounds of N with alkali, alkaline-earth, transition metal, and nonmetal elements has been recently synthesized under high-pressure–high-temperature (HP-HT) conditions, generated using LH-DAC, by the direct chemical reaction of the corresponding element with N_2_ or using suitable precursors like its azides.^5,7–16^ Based on *in situ* absorption of infrared laser light by a sample under high density,^17–20^ the LH-DAC enables simultaneous generation of pressure from few tenths up to hundreds of GPa and temperature of thousands of K, providing unique possibilities to open new reactive paths and explore regions of the potential energy surface of the system, which are inaccessible at ambient conditions.^21^

The energetic barriers preventing the direct nitridation reaction at ambient pressure can be thus overcome, allowing the synthesis of new stable or metastable reaction products, which are potentially recoverable to ambient pressure. Moreover, the DAC has shown extraordinary versatility of use in combination with a variety of advanced *in situ* probing techniques, enabling in-depth sample characterization.^17,20,22–28^

Beyond binary nitrides, in which N is bonded to a less-electronegative element, an entire class of new materials has emerged by the application of this synthetic method, revealing an astonishingly rich complexity in the HP chemistry of N compared to ambient pressure, with exotic N-species holding the stage in the HP domain: pernitrides units, pentazolate and hexazine rings, polynitrogen linear and branched chains, helices, macrocycles, and framework structures.^7,8,29^

Nevertheless, within this thriving context, excluding the α-P_3_N_5_ and γ-P_3_N_5_ polymorphs and unstable azides, no crystalline binary nitride of heavier pnictogens has been synthesized and convincingly characterized using ambient pressure methods. Moreover, the direct chemistry of N with heavier pnictogens in condensed phase has remained unexplored.^2–5,13,30,31^

In 2014, the experimental preparation of phosphorene from black phosphorus^32^ marked a turning point for group 15 elements in the family of xenes,^33^ drawing attention to their layered crystal structures^34,35^ as sources for corresponding single-layer materials. Thereafter, computational studies suggesting N-doping as a method to stabilize these 2D materials and tune their electronic, optical, and catalytic properties sparked interest in the chemistry of N with heavier pnictogens.^36–38^

Theoretical studies provided further insights, suggesting the existence of new compositional spaces for crystalline inorganic nitrides.^6^ They also indicated pnictogen nitrides specifically to match the most negative cohesive energy with the highest activation barrier for transforming the metastable state into the thermodynamically stable phase,^39^ and proposed new thermodynamic routes for their synthesis based on the exploitation of high chemical potential N.^40^

In this scenario, our idea that HP-HT conditions could effectively unveil an unexplored chemistry of heavier pnictogens with N in condensed phase, leading to the discovery of the missing crystalline pnictogen nitrides, stems from two apparently uncorrelated clues emerging from our studies on phosphorus. These studies highlight consistency in the structural and reactive properties of pnictogens not only within group 15, thus suggesting the formation of binary compounds of N with heavier pnictogens, but also compared to adjacent groups of the periodic table, for which crystalline nitrides have been indeed reported to form,^5,12,16,41,42^ adding focus on the importance of the electron lone pairs.

The first hint came from the experimental discovery of the pseudo simple-cubic (*p-sc*) structure in the phase diagram of phosphorus,^1,43^ which significantly raised the high-pressure limit for the existence of its layered structures up to ∼30 GPa, finally solving the anomaly of P, for which this value was lower than in As, and showing that this limit consistently decreases in group 15 with increasing the atomic number and with decreasing the strength of the *s-p* orbital mixing.^43^ Structural consistency was further supported a few years later by the HP-HT synthesis of a black-phosphorus-type structure in N (bP-N)^44,45^ and by the identification of a triple point in the phase diagram of P analogous to that of N under higher *pT* conditions,^46^ separating the molecular low-density liquid, the polymeric high-density liquid, and crystalline bP.^47^

The second hint came from the activation of a direct reaction between P and H_2_ under HP-HT conditions (1.2 GPa, ≲1000 K), leading to the synthesis of PH_3_, and from the discovery of the crystalline vdW compound (PH_3_)_2_H_2_.^48^ The direct synthesis of phosphine represents for P the long sought-after chemical reaction, which, in the case of N, is at the core of the Haber–Bosch process for the synthesis of ammonia from N_2_ and H_2_, thus reconnecting the high-pressure reactivity of P to that of lighter N. The discovery of (PH_3_)_2_H_2_, never before observed for any of group 15 elements, represents the missing tile for pnictogens in the family of isostructural X_2_H_2_ compounds (X = CH_4_, H_2_S, H_2_Se, HI) formed with H_2_ by the molecular hydrides of groups 14, 16 and 17, likewise suggesting the existence of missing group 15 nitrides by comparison with other groups.

## 3 HP-HT Chemistry of N_2_ with Heavier Pnictogens in LH-DAC

Pnictogens heavier than N do not react spontaneously with N_2_ under ambient conditions and even at high pressure along the room-temperature (room-*T*) isotherm up to 50–100 GPa, according to our experiments. Experimentally, our idea to induce chemical reactivity was based on the use of pressure, statically generated by membrane DACs, to increase the density and reduce the interatomic distances, and temperature, generated by laser-heating, to overcome the energetic barriers and open new reaction pathways. The solid pnictogen is used as a reactant and a laser absorber, whereas N_2_ is used as a reactant and a pressure transmitting medium. Pressure and temperature are then progressively increased to identify the reaction conditions, which are typically revealed by a sudden glowing of the sample, attesting for the effective heating, by a change in the sample aspect, and by the appearance of new Bragg peaks and vibrational bands.

Single-crystal and powder synchrotron X-ray diffraction (XRD) at the ID27 and ID15B beamlines of the European Synchotron Radiation Facility (ESRF) and FTIR^22^ and Raman^23^ spectroscopy at the European Laboratory for Non-linear Spectroscopy (LENS) were used to gain structural information and direct insight on bond cleavage and formation.

## 4 Crystalline Phosphorus Nitride

Until recently, only α-P_3_N_5_ (*Cc*, *Z* = 4) and γ-P_3_N_5_ (*Imm*2, *Z* = 2) were structurally characterized.^49,50^ α-P_3_N_5_ was synthesized from thermal decomposition of chemical precursors at ambient pressure,^49^ whereas γ-P_3_N_5_ was obtained by HT compression of partially crystalline α-P_3_N_5_.^50^

To our knowledge, the activation of HP-HT chemical reactivity between P and N_2_ in condensed phase, leading to direct synthesis of crystalline phosphorus nitride, was first reported in 2018 ^51^ and 2019,^52^ when preliminary XRD and Raman spectroscopy results on γ-P_3_N_5_ from our group were presented, providing the equation of state of the material and the evolution with pressure of its Raman spectra and vibrational frequencies.^2^

In our experiments, LH conditions were progressively increased, until a chemical reaction was observed at 9.1 GPa and 2000–2500 K, which, by extrapolating the melting line of P, correspond to the liquid phase (Figure 1A).

**Figure 1:**
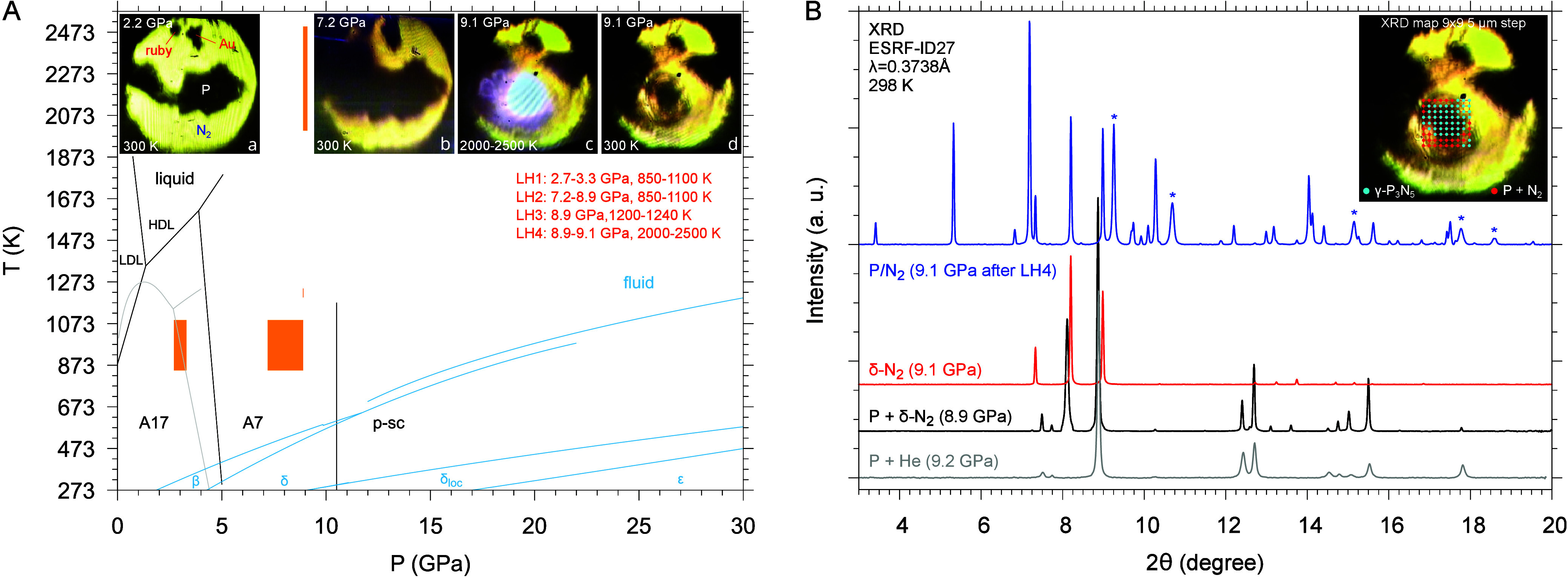
(A) Phase diagram of phosphorus (black^1,47^ and gray^59^) and nitrogen (blue)^2^ with laser-heating conditions (orange areas: LH1, LH2, LH3 and LH4).^2^ Photomicrographs of the sample: (a) after loading and before LH, (b) 7.2 GPa and ambient T after LH2 (7.2–8.9 GPa, 850–1100 K), (c) 9.1 GPa and 2000–2500 K during LH4, and (d) 9.1 GPa and ambient T after LH4. [Panel (A) has been adapted with permission from refs 2 and 47 . Copyright 2022, American Chemical Society, Washington, DC.] (B) XRD patterns acquired before LH2 in the dark (A7-P and δ-N_2_, black) and transparent (δ-N_2_, red) areas and after LH4 at 9.1 GPa in one of the blue points on the red mapping grid (9 × 9, 5 μm spacing) at the center of the laser-heated area (blue). The asterisks mark peaks from the Au pressure sensor. A diffraction pattern of P in He is displayed (gray) to help identify the A7-P peaks. Intensity was normalized to the most intense peak, and the background was subtracted. [Panel (B) has been adapted with permission from ref 2. Copyright 2022, American Chemical Society, Washington, DC.]

The XRD and Raman mapping of the sample after LH indicate that, whereas at the center of the laser-heated area the reaction product is γ-P_3_N_5_ (Figure 1B, 2C, 2E), in the outer regions of the laser-heated area, where the temperature is likely lower, the detection of specific Bragg peaks reveals the formation of α-P_3_N_5_ (Figure 2D, 2E), which appears as an intermediate step toward the progressive N-coordination of P with increasing coordination number (CN) from 4 to 5, in agreement to the pressure coordination rule.^50,53,54^ In terms of coordination polyhedra, whereas α-P_3_N_5_ consists of vertex- and edge-sharing PN_4_ tetrahedra (CN 4), γ-P_3_N_5_ is made of vertex-sharing PN_4_ tetrahedra (CN 4) and vertex- and edge-sharing PN_5_ square-pyramids (CN 5), resulting in a 32% higher density compared to α-P_3_N_5_.

The high-pressure structural behaviors of α-P_3_N_5_ and γ-P_3_N_5_ have been further studied (Figure 2). For γ-P_3_N_5_ XRD, Raman spectroscopy and DFT calculations during room-*T* compression and decompression unveiled a characteristic anisotropic compressibility, originating from the stiffness of the connection framework of the square-pyramids along the *b*-direction (Figure 2B).^2^

**Figure 2:**
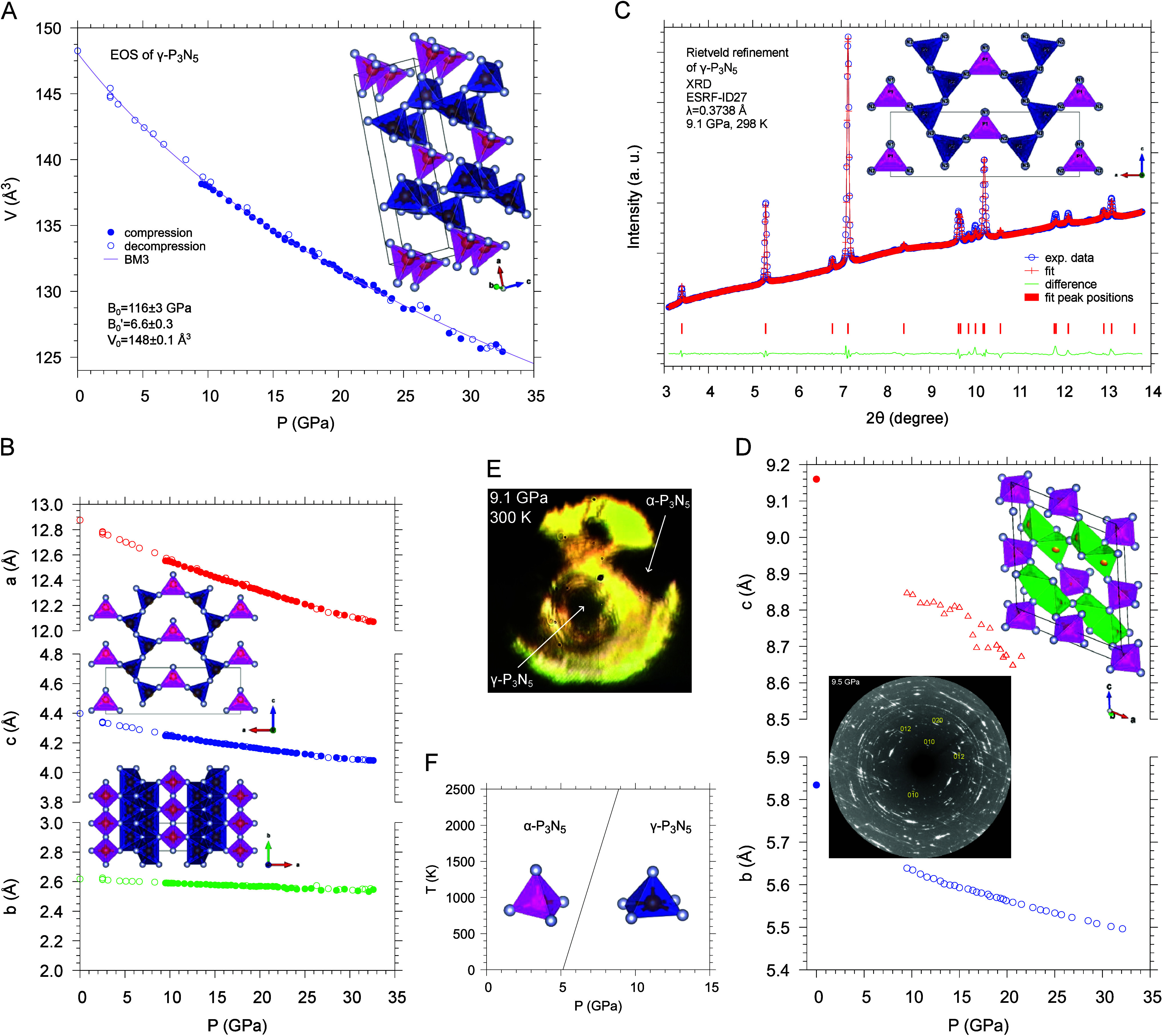
(A) Equation of state and crystal structure of γ-P_3_N_5_ with highlighted unit cell. [Legend: PN_4_ tetrahedra, magenta; PN_5_ square-pyramids, blue.] (B) Pressure dependence of the lattice parameters of γ-P_3_N_5_. (C) Rietveld refinement of an XRD pattern acquired at the center of the laser-heated area corresponding to γ-P_3_N_5_. (D) Pressure dependence of the *b* and *c* lattice parameters of α-P_3_N_5_. [Legend: PN_4_ tetrahedra, vertex-sharing, magenta; vertex- and edge-sharing, green.] Filled circles values at ambient pressure are from ref 49. The diffraction image highlights the reflections detected in the outer region of the laser-heated area (panel (E)) assigned to α-P_3_N_5_. (E) Sample image after LH4. (F) Calculated equilibrium pressure as a function of temperature for the α-P_3_N_5_ to γ-P_3_N_5_ phase transition associated with a 4 → 5 increase in the CN of P by N.^2^ [Panels (A)–(F) have been adapted with permission from ref 2. Copyright 2022, American Chemical Society, Washington, DC.]

These studies demonstrated, for the first time, the effectiveness of HP-HT conditions generated by LH-DAC to induce direct chemical reactivity between P and N_2_, leading to the synthesis of different crystalline P_3_N_5_ polymorphs, depending on the applied conditions, and paving the way for the exploration of crystalline nitrides of heavier pnictogens.

Indeed, in 2021, another study confirmed our findings about γ-P_3_N_5_ and, while exploring a higher pressure range, obtained another reaction product by further compression and laser-heating of γ-P_3_N_5_ in N_2_ at 70 GPa.^55^ The reaction product was not identified, but insight on a different chemistry at higher pressure was given, as suggested by theoretical predictions.^56,57^ One year later, two ultra-incompressible denser phosphorus nitride structures (*C*2/*c* δ-P_3_N_5_ and pyrite-type *Pa*3̅ PN_2_), containing octahedrally coordinated phosphorus, were synthesized at higher pressure by another group,^58^ using exactly the same method that we first reported for α- and γ-P_3_N_5_.

## 5 Crystalline Arsenic Nitride

Taking advantage of our results on P, we used the LH-DAC to explore the HP-HT chemical reactivity of As and N in the condensed phase. No reactivity was observed at 14.7 GPa and 1400 K, whereas a chemical reaction was detected when laser-heating was performed after increasing pressure above 25.0 GPa (Figure 3A).^3^ The analysis of the diffraction data allowed identification of single-crystal domains, indicating the formation of a reaction product having AsN stoichiometry and crystal structure belonging to cubic space group *P*2_1_3 (*Z* = 32). As far as we know, this structure, which was determined for different pressures at room T between 30 and 40 GPa, represents the first report of the synthesis of crystalline arsenic nitride,^3^ according to the following chemical equation:

$$\mathrm{As}+\frac{1}{2}N_{2}\overset{P>\mathrm{25.0 GPa}}{\underset{T>\mathrm{1400 K}}{\to }}\mathrm{AsN}$$ 1

**Figure 3:**
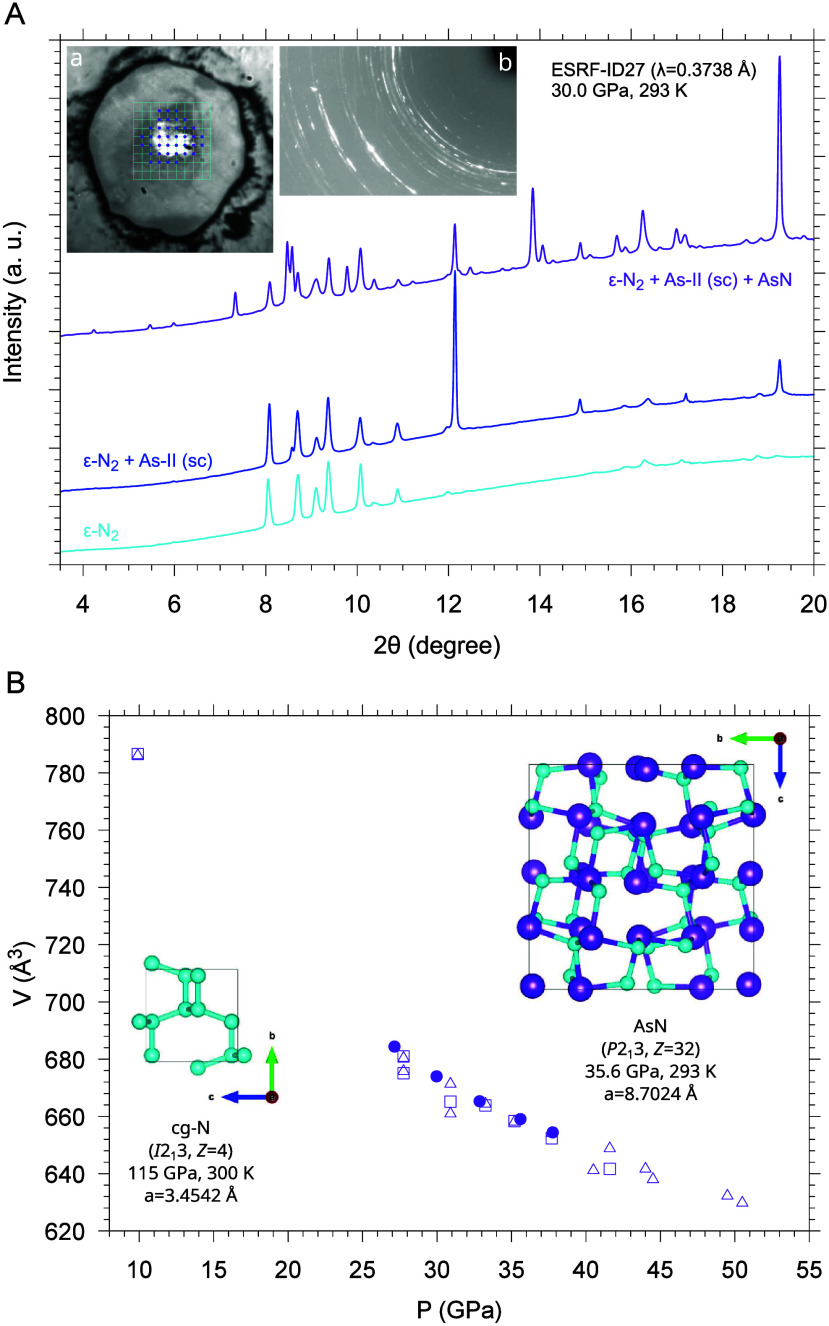
(A) XRD patterns were acquired at room temperature and 30 GPa on a sample laser-heated at 25.0 GPa. The patterns were acquired in the points of the mapping grid (10×10, 5 μm spacing) superimposed to the sample image (a), where the shining central area corresponds to the original piece of As surrounded by N_2_. The violet full circles on the grid indicate the sample points at the center of the laser-heated area where AsN was detected (violet trace and detector image (b)), whereas the outer unlabeled grid points, at the margins of the laser-heated area, indicate patterns where only ϵ-N_2_ (cyan trace) or ϵ-N_2_ and *sc*-As (blue trace) were observed. [Reproduced with permission from ref 3. Copyright 2022, Wiley–VCH GmbH.] (B) Room-*T* pressure dependence of the unit-cell volume of AsN. Data were acquired on different samples synthesized at 36.0 GPa (solid symbols, single-crystal XRD) and 25.0 GPa (empty symbols denote powder XRD: squares and triangles respectively refer to the *hkl* = 111 and *hkl* = 211 reflections used to derive the lattice parameters). The AsN^3^ and cg-N^61^ unit cells, scaled to their real relative size, are shown (As violet, N cyan). [Adapted with permission from ref 3. Copyright 2022, Wiley–VCH GmbH.]

The analysis of bond distances and angles indicates that the connection scheme of AsN is made of a 3D framework of alternating vertex-sharing AsN_3_ and NAs_3_ trigonal pyramids, where only single As–N bonds are present. Every As is covalently bonded to three N and vice versa, with As and N respectively featuring oxidation states +3 and −3 and hosting a nonbonding electron lone pair (LP) at the vertex of the corresponding trigonal pyramids (Figure 4). LPs may also be pictured as occupying the fourth vertex of LP-AsN_3_ and LP-NAs_3_ pseudotetrahedra.^60^

**Figure 4:**
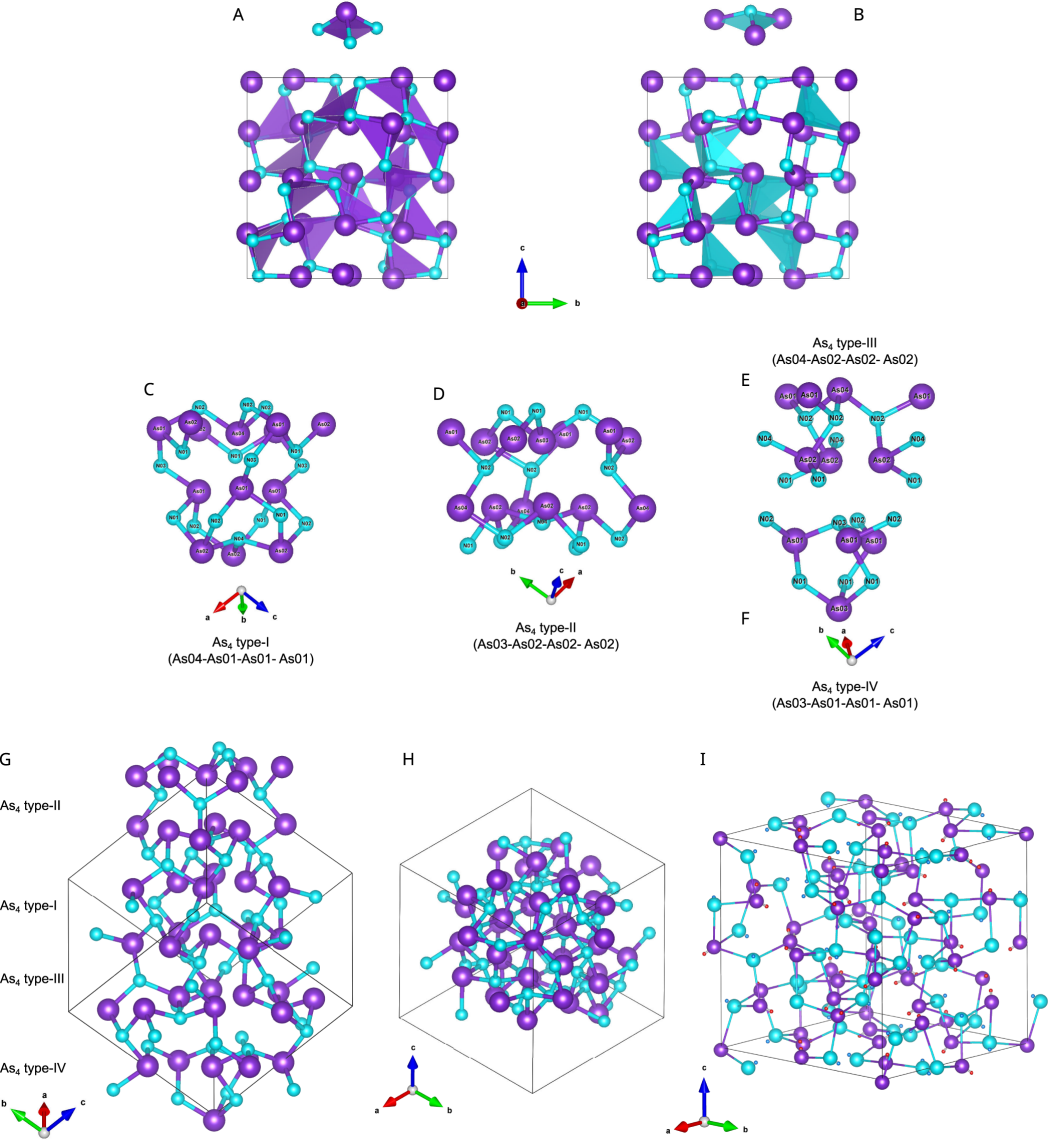
Views of the *P*2_1_3 unit cell of AsN at 35.6 GPa and 293 K (As violet, N cyan) highlighting the AsN_3_ (A) and NAs_3_ (B) trigonal pyramids and selected regions of the crystal structure showing the four types of As_4_ tetrahedra (panels (C)–(F)) and their arrangement along the *C*_3_ axis (panels (G) and (H)).^3^ [Panels (A) and (B) have been reproduced and panels (C)–(H) have been adapted with permission from ref 3. Copyright 2022, Wiley–VCH GmbH.] The localization of the LPs for a calculated structure of AsN at 30 GPa is also shown (I: As-LP red, N-LP blue).^60^ [Panel (I) has been adapted with permission from ref 60. Copyright 2024, American Chemical Society, Washington, DC.]

This atomic arrangement determines the presence of high-electron-density regions in the unit cell of AsN, where LPs interact with each other through strong highly directional repulsive interactions. The relative orientations of the stereochemically active LPs cooperatively play a key role in stabilizing the crystal structure of AsN by minimizing the repulsive energy of such interactions.

This occurrence is indirectly revealed also by the evolution with pressure of bond distances and angles, and by the presence of characteristic structural motifs driven by the relative orientation of the stereochemically active LPs, such as cages, cavities, and four types of As_4_ tetrahedra (I–IV), where the As atoms are not directly bonded but bridged by at least one N atom (Figure 4).

These structural features have been thoroughly characterized by DFT calculations through a detailed analysis of the atom–atom, LP–LP, and atom–LP distances.^60^ Our calculations have found the *P*2_1_3 structure of AsN to be stable with respect to crystalline As and N_2_ for pressures higher than 17 GPa, indicating AsN to be stable at the synthesis conditions and metastable below 17 GPa.

The analysis of the calculated vibrational modes revealed the presence of highly localized phonon modes, which are associated with the modulation of the LP electron density of As and N atoms located on the *C*_3_ axis along the [111] crystallographic direction. This feature suggests potential implications for low thermal conductivity, which deserve further investigation. Our calculations indicate AsN to be a semiconductor with a 2.51 eV indirect electronic bandgap at 30 GPa.^60^

## 6 Crystalline Antimony Nitride

No crystal structure of antimony nitride had been experimentally synthesized and conclusively characterized, although insights were provided by recent experimental^62^ and theoretical^63^ studies. In our experiments, no reactivity between Sb and N_2_ was observed under room-*T* compression in the explored pressure range and when compressing the sample to 15 GPa and laser-heating to 1600 K. A chemical reaction was instead repeatedly observed when compressing Sb in N_2_ at 32–35 GPa and laser-heating between 1600 and 2200 K, where Sb^64^ and N_2_ ^46^ are liquid and fluid, respectively (Figure 5A).

**Figure 5:**
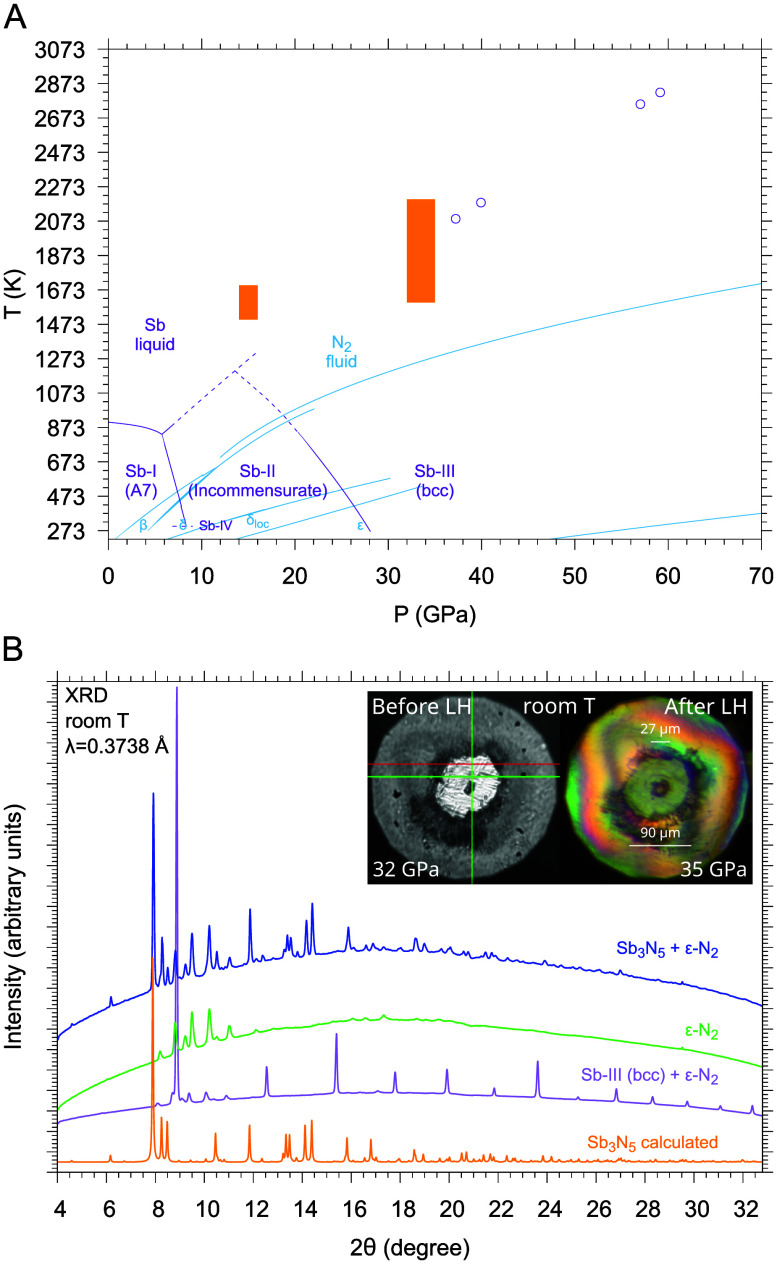
A. Phase diagrams of Sb (violet)^64^ and N (blue)^2^ with LH conditions (orange areas). B. Room T XRD patterns of a sample containing Sb and N_2_ acquired at 32 GPa in the center of the sample before LH, indicating the presence of crystalline bcc Sb-III and ϵ-N_2_ (purple); at 35 GPa in the transparent region after LH, indicating the presence of ϵ-N_2_ (green); and at 35 GPa in the center of the laser-heated area after LH, indicating the presence of a crystalline reaction product and excess ϵ-N_2_ (blue). The XRD pattern of Sb_3_N_5_ (*Cmc*2_1_) calculated from our single-crystal data is also shown (orange). The photomicrographs show the change in the aspect, size and shape of the starting piece of Sb-III after LH. [Panel (B) has been adapted with permission from ref 4. Copyright 2024, Wiley-VCH GmbH.]

The XRD mapping of the sample at room T after LH (11×11 grid, 3 μm spacing) indicated quantitative consumption of Sb in excess N_2_ upon LH. The disappearance of the Bragg peaks of Sb–III is associated with the appearance of new peaks incompatible with any known structure of Sb or N (Figure 5B). The analysis of single-crystal domains indicated the formation of a reaction product with Sb_3_N_5_ stoichiometry and a crystal structure belonging to orthorhombic space group *Cmc*2_1_ (*Z* = 4). To our knowledge, this structure represents the first evidence for the synthesis of crystalline antimony nitride via the following chemical equation:

2 $$3\mathrm{Sb}+\frac{5}{2}N_{2}\overset{P>\mathrm{32 GPa}}{\underset{T=\mathrm{1600-2200 K}}{\to }}{\mathrm{Sb}}_{3}N_{5}$$

Sb_3_N_5_ features Sb in the +5 oxidation state and N in the −3 oxidation state. The connection scheme contains only covalent Sb–N bonds, without formation of azide, diazenide and pernitride units or polynitrogen chains as instead suggested by literature calculations.^63^ Two Sb (Sb01, Sb02) and three N (N01, N02, and N03) symmetry independent atoms are present in the asymmetric unit.

Considering a 2.50 Å cutoff for the Sb–N bond length, the crystal structure of Sb_3_N_5_ can be described in terms of coordination polyhedra as made of octahedral and trigonal-prismatic SbN_6_ units (CN 6), respectively involving Sb01 and Sb02 (see Figures 6A, 6C, and 6E–G). Among N atoms, N01 and N02 exhibit distorted tetrahedral coordination (Figures 6J and 6K), whereas N03 exhibits a trigonal-pyramidal coordination, underlying the presence of an electron lone pair (Figure 6L), similarly to δ-P_3_N_5_.^58^ However, considering all of the Sb–N distances, two additional N03 atoms are located at 2.54 Å from Sb02, revealing weaker secondary bonding interactions, possibly becoming stronger at higher pressure. This occurrence determines an increase in the CN of Sb02 from 6 to 8, leading to SbN_8_ units in square-antiprismatic coordination (SAPR-8) (see Figures 6B, 6D, 6H, and 6I), and in the CN of N03 from 3 to 4, leading to distorted tetrahedral coordination (Figure 6M).

**Figure 6:**
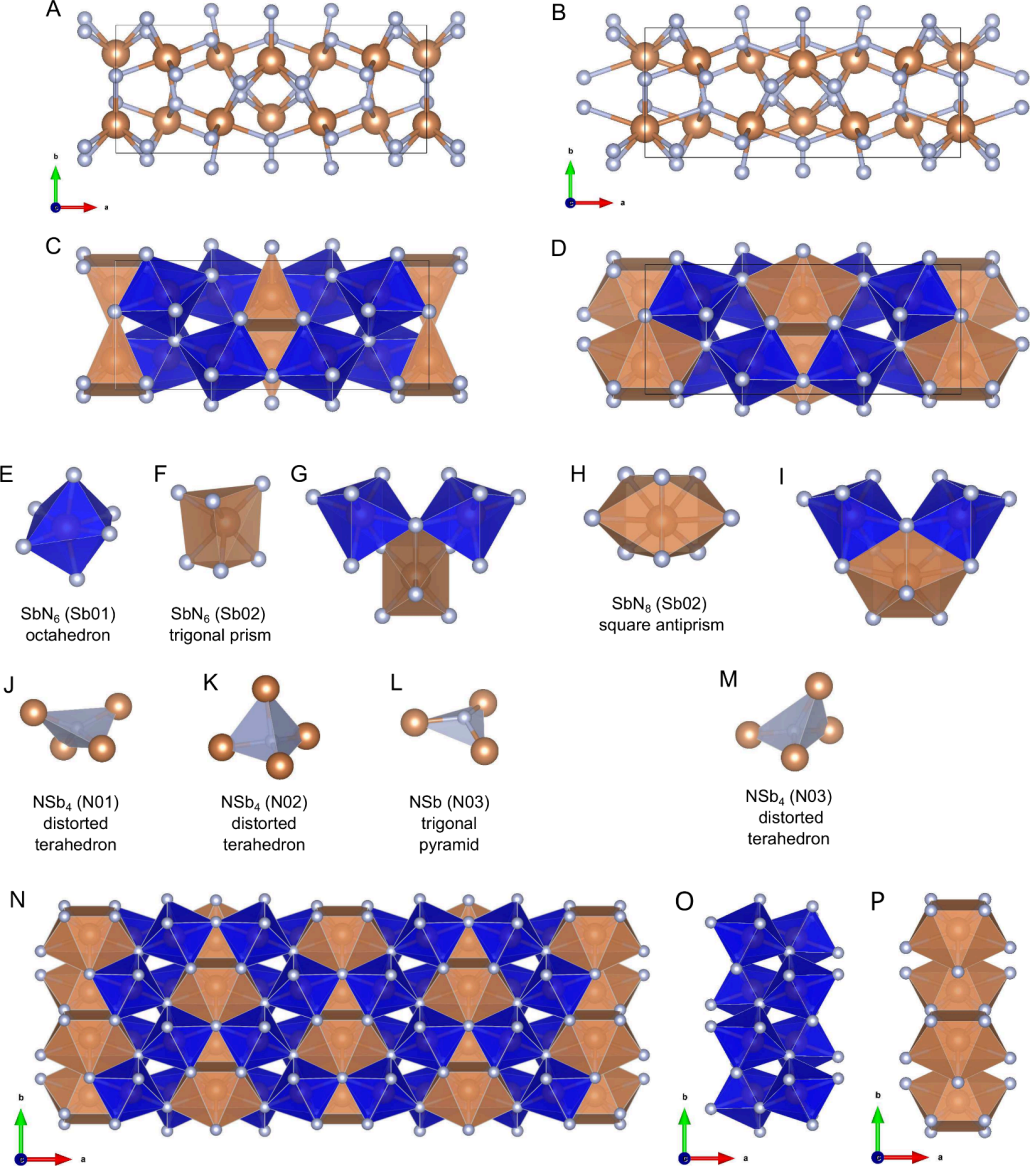
(A–D). Unit cell of Sb_3_N_5_ in the ball–stick (panels (A) and (B)) and polyhedra (panels (C) and (D)) representations (Sb orange, N gray) adopting a 2.50 Å cutoff value for the Sb–N bond length (panels (A) and (C)) and considering all the Sb–N distances (panels (B) and (D)). (E-M). Coordination polyhedra resulting from the 2.50 Å cutoff value (panels (E)–(G) and (J)–(L)) and considering all the Sb–N distances (panels (E), (H)–(K) and (M)). Panels (G) and (I) highlight the two additional interactions between Sb02 and the LPs of two N03 atoms. (N-P). Alternated stacking of double-layers of SbN_6_ octahedra (panel (O), Sb01) and single-layers of SbN_8_ square-antiprisms/SbN_6_ trigonal-prisms (panel (P), Sb02). [Panels (A)–(D), (G), (I) and (N)–(P) have been reproduced and panels (E), (F), (H) and (J)–(M) have been adapted with permission from ref 4. Copyright 2024, Wiley-VCH GmbH.]

Overall, the Sb_3_N_5_ structure can be described as an ordered stacking in the *bc* plane of bilayers of vertex-sharing SbN_6_ octahedra involving the Sb01 atoms, alternated to monolayers of vertex- and edge-sharing SbN_6_ trigonal-prisms or SbN_8_ square-antiprisms involving Sb02 (Figures 6N–P).

## 7 Crystalline Bismuth Nitride

No structural characterization of bismuth nitride was reported until recently, when the structures of two crystalline polymorphs were determined: *Pbcn* BiN was synthesized by direct reaction of Bi and N_2_ under HP-HT conditions in LH-DAC (42.5 GPa, > 1800 K), whereas *Pca*2_1_ BiN was obtained during room-*T* decompression of *Pbcn* BiN below 12.5 GPa and down to ambient pressure.^31^ Both compounds contain two types of Bi atoms with three N nearest neighbors, but in *Pbcn* BiN one Bi atom peculiarly approaches CN 4 below 51.0 GPa. Overall, despite no space group relation, the structures of BiN exhibit similarities to AsN,^4^ rather than to the structures of P^2,49,50,55,58^ or Sb^4^ nitrides. Interestingly, space group-subgroup relations connect *P*2_1_3 AsN to *I*2_1_3 cg-N and *Pca*2_1_ and *Pbcn* BiN to *Cmce* bP and bP-N.^31^

## 8 Discussion, Conclusions, and Perspectives

While HT is critical for the activation of chemical reactivity between N_2_ and heavier pnictogens, the reduction of interatomic distances determined by pressure emerges as a key factor for the formation of the crystalline pnictogen nitrides.

According to the available data the minimum reaction pressure of P (9 GPa),^2,55^ As (25 GPa),^3^ Sb (32 GPa),^4^ and Bi (42.5 GPa)^31^ with N_2_ increases with the pnictogen atomic number.

Moreover, our experiments on As^3^ and Sb,^4^ where comparable HT was generated at different pressures, suggest the existence of a threshold pressure along a given isotherm for the formation of the corresponding crystalline nitride. Laser heating below this pressure, although leading to pnictogen melting, is apparently ineffective at triggering a chemical reaction.

According to the phase diagrams available in the literature^47,64–66^ and our unpublished data, the HP-HT reactivity of P, As, Sb, and Bi with N_2_ appears to take place in the liquid phase, where the higher atomic mobility likely favors the pnictogen coordination by N and the arrangement of the corresponding polyhedra in the crystal structures of the reaction products.

The reactions proceed quantitatively upon LH in excess N_2_ until the complete consumption of the other pnictogen, and the crystalline products persist at room T and HP once LH is switched off, allowing further compression and decompression studies.

No phase transitions have been reported so far on room-*T* compression of the discovered crystalline pnictogen nitrides in the investigated pressure ranges, while persistence on decompression below the pressure of synthesis is typically observed, in agreement with the theoretically suggested metastability of crystalline pnictogen nitrides, due to the kinetic barriers imposed by the strong chemical bonds and high cohesive energy, which are able to lock unfavorable atomic arrangements.^39^ This occurrence is supported by the detection of α-P_3_N_5_ up to 32.6 GPa and γ-P_3_N_5_ up to 45.5 GPa upon room-*T* compression far above their predicted stability ranges,^2,55,67^ by the recovery of γ-P_3_N_5_ ^2,55^ and δ-P_3_N_5_,^58^ respectively, to ambient pressure and 7 GPa and by the persistence of AsN down to 9.8 GPa,^3^ below its calculated stability pressure of 17 GPa.^60^ It is also further attested by the observation of two crystalline polymorphs of P and Bi nitrides (*P*2_1_/*c* α′-P_3_N_5_ and *Pca*2_1_ BiN), obtained during decompression of the corresponding high-pressure phases (δ-P_3_N_5_ and *Pbcn* BiN) and persisting at ambient pressure,^31,58^ which highlights the role of the decompression path in the synthesis of new materials.

The HP-HT conditions of synthesis evidently determine the pnictogen CN in the case of phosphorus, for which a broader dataset is available. Moreover, in analogy to the HP-HT α-P_3_N_5_ → γ-P_3_N_5_ phase transition,^2,50^ the similarity of the Raman spectra acquired after laser-heating γ-P_3_N_5_ in N_2_ at 67–70 GPa^55^ with those acquired on δ-P_3_N_5_, synthesized by laser-heating P in N_2_ at 72 GPa,^58^ indicates γ-P_3_N_5_ as an intermediate step in the progressive N-coordination of P toward δ-P_3_N_5_ and the pressure coordination rule to be valid not only for the α-P_3_N_5_ → γ-P_3_N_5_ transformation, but also for the γ-P_3_N_5_ → δ-P_3_N_5_ one at higher pressure,^50^ respectively involving 4 → 5 and 5 → 6 increases in phosphorus CN.

Only one crystalline nitride polymorph has been experimentally synthesized and characterized so far for As^3^ and Sb.^4^ However, the experimental observation of two additional longer distances (Sb02–N03) in Sb_3_N_5_, expanding the coordination sphere of Sb02 from 6 to 8, suggests the strengthening of weaker bonding interactions and the stabilization of high CNs of Sb with increasing pressure. Although not mentioned by the authors, two longer Sb–N distances, extending the CN of Sb from 6 to 8, are found also in calculated Sb_2_N_3_ (*Cmcm*) at 120 GPa.^4,63^ An even higher CN of Sb by N has been predicted at 100–140 GPa in SbN_6_ (*R*3̅*m*), featuring icosahedral SbN_12_ coordination.^68^ The effect of pressure in stabilizing high CNs of Sb­(V) by N is also consistent with the fact that, despite CN 4 in unusual tetrahedral coordination being recently reported in Zn_2_SbN_3_ ^69^ and Mg_2_SbN_3_ ^70^ at ambient conditions, no CN 4 is observed in Sb_3_N_5_ at 35 GPa, and with the fact that CN 6 in octahedral coordination has been observed also in HP-HT synthesized SbCN_3_.^71^

Noticeably, the discovery of crystalline Sb_3_N_5_ unveiled the existence of trigonal/square-antiprismatic pnictogen coordination by N,^4^ while confirming the tetrahedral coordination of N, first reported in δ-P_3_N_5_ among binary group 15 nitrides.^58^

Whereas Sb_3_N_5_ exhibits the same stoichiometry and shares structural similarities with δ-P_3_N_5_, AsN unveiled a completely different HP-HT chemistry of As with N compared to P and Sb, in terms of valence, stoichiometry and crystal structures, rather exhibiting analogies with the HP-HT behavior of N. AsN (*P*2_1_3)^3^ appears indeed closely related to cg-N (*I*2_1_3),^61^ as its structure can be ideally obtained by replacing every nearest N in cg-N with As.

However, whereas cg-N was synthesized under higher *pT* conditions and could be decompressed at room *T* only down to 42 GPa, the unit cell of AsN persists on room-*T* decompression at least down to 9.8 GPa (Figure 3B). Therefore, considering the interest in recovering high energy-density cg-N for envisaged applications as explosive and green propellant in aerospace engineering and sustainable mobility,^72^ understanding the effects underlying the stability of AsN may open new perspectives for the HP synthesis of new advanced materials of energetic and technological relevance, potentially recoverable to ambient conditions, as suggested by doping of polynitrogen materials.^54,73–75^

Another insight emerging from the discovery of AsN, which further highlights the importance of the LPs in the HP chemistry of pnictogens, consistently with the mechanism of interlayer bond formation in P^1,43^ and the stabilization of crystalline (PH_3_)_2_H_2_,^48^ is related to the fact that, whereas cg-N features *all-gauche* conformation of the stereochemically active LPs about the N–N bonds (LP–N–N–LP), among the eight dihedral angles (LP–As–N–LP) in the unit cell of cubic AsN, two exhibit *anti* conformation and six *gauche* conformation of the LPs, with respect to the corresponding As–N bond.^60^

Furthermore, the experimental observations of bP-N,^44^ in which 2/3 of the dihedral angles show *gauche* and 1/3 *anti* conformation, and of hexagonal-layered-polymeric N (HLP-N),^76^ in which the dihedral angles exhibit a mixture of LP-*eclipsed*, *eclipsed*, and *gauche* conformations, suggest the potential existence of other structures featuring different LPs conformation ratio also in the As/N system under different HP-HT conditions.

As the properties of crystalline pnictogen nitrides are concerned, the strong chemical bonds, compact octahedral hexa-coordination and specific connection scheme of the PN_6_ octahedra in δ-P_3_N_5_ and PN_2_ make them ultraincompressible materials appealing for mechanical applications,^58^ while the characteristic bonding framework make α-P_3_N_5_ and γ-P_3_N_5_ wide bandgap semiconductors exhibiting linear and nonlinear optical properties, with γ-P_3_N_5_ featuring birefringent phase-matched second harmonic coherent output down to 286 nm.^67,77^

The LP localization observed in AsN appears as a highly desirable chemical feature to enable specific properties in the design and HP synthesis of new advanced materials of high technological relevance, including thermoelectric, photovoltaic, ferroelectric, and topological materials with implications for spintronics, catalysis, and quantum information science,^78^ as recently suggested by a theoretical study predicting three stable structures in the As/N system between 10 and 30 GPa and specifically proposing layered *Cmc*2_1_ AsN to exhibit bulk photovoltaic effect.^79^

The HP-stabilization of N-coordinated Sb in its highest oxidation state, which has been reported to enable compelling optoelectronic properties and bandgap engineering at ambient pressure,^69,70,80^ emerges as a powerful innovative tool for the synthesis of advanced materials under extreme conditions.^4,63,68,81^ Understanding the stability/mestastability of the Sb–N system in terms of structure and composition is technologically relevant for the synthesis of phase change memory and thermoelectric materials^4^ and of semiconductors for applications in optoelectronics, photovoltaics and photoelectrochemical cells.^69,70,80^ Furthermore, the HP structures of calculated SbN_4_ ^63^ and experimentally synthesized Sb_3_N_5_ ^4^ have been proposed as high energy-density materials,^63,81^ while predicted SbN_6_ has been indicated as a superconductor.^68^

Additional interest concerns the intriguing properties predicted for 2D single-layer polymorphs of N with heavier pnictogens,^4,37,38^ whose 3D structures may possibly emerge from HP-HT studies.^63,79^

Beyond the obvious relevance for the advancement of fundamental chemical knowledge, the complex pattern of differences and similarities emerging from the newly discovered nitrides of group 15 elements clearly remarks our limited knowledge about this family of compounds, requiring further investigations not only to account for their properties, but also to explore the existence of other structures, stoichiometries, and atomic coordinations, including complex structures like those formed by N with other elements at high pressure.

The results presented here demonstrate that HP-HT conditions generated by LH-DAC are extremely effective in activating direct chemical reactivity between N_2_ and heavier pnictogens, marking a breakthrough in the synthesis and exploration of elusive crystalline pnictogen nitrides. Following the direct synthesis of α-P_3_N_5_ and γ-P_3_N_5_, the discoveries of crystalline AsN and Sb_3_N_5_ represent milestones in the chemistry of group 15 elements as they provide the long sought-after experimental evidence of As and Sb, beyond P, to form crystalline nitrides, filling a gap about the existence of these compounds for the fourth and fifth period under group 15 ^5,30^ and paving the way to the exploration of an entire class of new advanced pnictogen-based materials of chemical, energetic, and technological relevance.
